# Heat Shock Protein 70 Acts as a Potential Biomarker for Early Diagnosis of Heart Failure

**DOI:** 10.1371/journal.pone.0067964

**Published:** 2013-07-09

**Authors:** Zongshi Li, Yao Song, Rui Xing, Haiyi Yu, Youyi Zhang, Zijian Li, Wei Gao

**Affiliations:** Department of Cardiology, Peking University Third Hospital and Key Laboratory of Cardiovascular Molecular Biology and Regulatory Peptides, Ministry of Health, Key Laboratory of Molecular Cardiovascular Sciences, Ministry of Education and Beijing Key Laboratory of Cardiovasicular Receptors Research Beijing, China; National Institute of Nutrition, India

## Abstract

Early identification for heart failure (HF) may be useful for disease modifying treatment in order to reduce heart disease progression or even to reverse it. In our previous studies, we have revealed a group of heat shock proteins (HSPs) which might be related to neonatal rat cardiomyocyte hypertrophy by proteomic approach. Here, we confirm that HSPs, including HSP27 and HSP70, altered in the early stage of cardiac remodeling in vivo animal model. Furthermore, plasma concentrations of those HSPs and their potential screening value were evaluated at different stages in 222 patient subjects. Plasma HSP27, HSP70 and HSP90 were measured using enzyme-linked immunosorbent assay. Results indicate that HSP70 was positively correlated to the severity (progression) of HF (r = 0.456, p<0.001). The area under the rate of change (ROC) curve was 0.601 (p = 0.017) in patients with stage B HF and 0.835 (p<0.001) in those with stage C HF. However, HSP27 and HSP90 did not display significant changes in any stage of HF in this study. Taken together, plasma concentrations of HSP70 elevated with the progression of HF and might act as a potential screening biomarker for early diagnosis of HF.

## Introduction

Cardiovascular diseases (CVDs) are major causes of mortality in the world. As a final stage of CVDs, heart failure (HF) becomes more prevalent year by year. It is therefore very important for the early warning, early diagnosis and treatment of heart failure. In 2005 the American College of Cardiology (ACC)/American Heart Association (AHA) updated their guidelines for the management of chronic HF (CHF) and identified four key stages of HF in patients (Hunt et al. 2005). Among patients without signs or symptoms of HF, those at high risk of HF were defined as stage A, and those who had structural heart disease were designated as stage B. Stage C included patients with current or past symptoms of HF, and stage D designated patients with truly refractory HF. Due to the lack of a screening strategy for detection, when diagnosed HF in hospital, the patients had always developed into the stage C or even stage D. An adequate screening test for early detection of stage B patients might greatly improve HF survival.

Originally demonstrated in Drosophila, the induction of heat shock proteins (HSPs) in response to elevated temperature is observed in many organisms, ranging from prokaryotic bacteria such as Escherischia coli to mammals including human. HSPs are constitutively expressed, but expression is up-regulated during pathophysiological stress [1]. During the last years, more and more information has become available on the specific role of individual heat shock proteins in protection of heart. Early in 1988, Currie et al. for the first time reported that whole body heat shock in rats is associated with improvement of cardiac functional recovery after a global ischemic insult, applied 24 h later [2]. Under physiological cardiac hypertrophy, heat shock improves the ischemic tolerance of the hypertrophied rat heart which related to the protection of HSP [3]. Niizeki T et al. found that serum HSP 60 level was related to the severity of CHF and associated with a high risk for late stage cardiac events in patients CHF [4]. However, so far the role of HSP in the early stage of heart failure is still unknown.

In our previous studies, we have revealed a group of HSPs which might be related to neonatal rat cardiomyocyte hypertrophy by proteomic approach [5], [6]. Here we have confirmed the expression disorder of HSPs in animal cardiac hypertrophy model. Cardiomyocyte hypertrophy always happens in the early stage of chronic heart failure (CHF). Chronic heart failure is characterized by central and peripheral abnormalities. The myocardium is continually exposed to stressors such as free radicals [7]. Immune activation is also thought to contribute to disease progression [8], and is associated with an adverse prognosis [9]. So far we still have no effective methods in identification of early stage of heart failure. Due to the importance of the early diagnosis and treatment of heart failure, we sought to characterize the expression of circulating HSP in CHF and evaluate the relationship between serum levels of this molecule with disease progression in patients with this condition.

## Materials and Methods

### Animals

All study protocols conformed to the Animal Management Rules of China (Documentation No. 55, 2001, Ministry of Health, China). All experiments were approved by the Committee for Ethics of Animal Experiments and were conducted in accordance with the Guidelines for Animal Experiments, Peking University Health Science Center. Ten-week-old male BALB/c mice were obtained from the Animal Department of the Peking University Health Science Center.

The isoproterenol-induced cardiac hypertrophy was carried out by infusing isoproterenol (Sigma, St Louis, Mo) in vivo using subcutaneously implanted micro-osmotic pumps (Alzet DURECT, Cupertino, CA). In brief, 10-week-old male Balb/c mice were anesthetized and implanted with micro-osmotic pumps and isoproterenol was delivered continuously at a rate of 5 mg/kg/day. The control mice received vehicle (100 µmol/L ascorbic acid). After 3, 7, 14 and 84 days, the mice were sacrificed by cervical dislocation after deep anesthesia with 2% isoflurane and the ratios of heart weight to body weight (HW/BW) and tibia length (HW/TL) were determined.

Echocardiographic analysis Cardiac hypertrophy and function were assessed by echocardiography before and after isoporterenol infusion using a Vevo 770TM Imaging System (VisualSonics Inc., Toronto, Canada) equipped with a 30-MHz microprobe under anesthesia with 1.5% isoflurane allowing spontaneous breathing.

### Western Blotting

Cell samples were lysed in 150 mL buffer containing 20 mM Tris-HCl (pH 7.4), 100 mM NaCl, 10 mM sodium pyrophosphate, 5 mM EDTA, 50 mM NaF, 1 mM sodium vandate, 0.1% SDS, 10% glycerol, 1% Triton X-100, 1% sodium deoxycholate, 1 mM leupeptin, 0.1 mM aprotinin and 1 mM PMSF. Protein concentration was estimated with a BCA protein assay kit (Pierce Chemicals). Proteins were separated on 10% SDS-polyacrylamide gels and then electrophoretically transferred to nitrocellulose membranes (Hybond, Amersham Biosciences, Uppsala, Sweden). The sheets were analyzed with antibodies according to the supplier’s protocol and visualized with peroxidase and an enhanced-chemiluminescence’s system (ECL kit, Pierce Chemicals). The following antibodies were used in this study: anti-HSP 27 (Santa Cruz Biotechnology, CA, USA, sc-9012), anti-HSP90 (Santa Cruz Biotechnology, sc-7947), anti-prohibitin(Abcam, Cambridge, UK) anti-HSP 70 (Santa Cruz Biotechnology, sc-32239), anti-eIF-5 (Santa Cruz Biotechnology, sc-28309). The films were then scanned with a calibrated densitometer and images were processed by PDQuest software.

### Study Population

All studies were approved by the Committee for Ethics of Peking University Health Science Center. Each patient who was enrolled in this study has signed the informed consent.

Patients were selected from the department of cardiology at the Peking University Third Hospital (Beijing, China) over the period July 2007 to April 2011. A total of 222 participants were divided into four groups composed of the different stages of HF [10].

Stage A (n = 76) included hypertensive patients (n = 56) according to the 2007 European Society of Hypertension (ESH)/European Society of Cardiology (ESC) hypertension guidelines [11] and stable angina pectoris patients (n = 20) diagnosed according to ESH/ESC 2006 guidelines [12].

Stage B (n = 68) included patients with old myocardial infarction (OMI, n = 46) or those who had structural heart disease (n = 22) [10]. OMI patients complied with the universal definition of myocardial infarction [13]. Structural heart disease was confirmed with echocardiographic assessment.

Stage C (n = 40) recruited previously symptomatic and currently asymptomatic CHF patients according to the ACC/AHA guideline [10].

The control (Cont) group (n = 38) included healthy volunteers without HF risk factors, cardiac structural lesion or HF symptoms.

Patients with acute myocardial infarction during the preceding 12 weeks, impairment of renal function or liver function, systemic inflammatory disease, infectious disease, cancer, acute cerebral infarction, pregnancy or who were using steroids that might affect the HSPs’ level were excluded.

### Biochemical Analysis

Fasting blood samples were drawn from antecubital vein of the patients and collected into evacuated EDTA tubes in the morning. Plasma samples were obtained within 30 min of collection, by centrifugation at 3000 g for 15 min at 4°C. To avoid repetitive freeze-and-thaw cycles, each sample was divided into seven to nine aliquots, immediately frozen, and stored at −80°C for analysis. Plasma HSP27, HSP70, HSP90 levels were determined by sandwich enzyme-linked immunosorbent assay (ELISA) with commercially available kits (RB Co., USA) according to the manufacturer’s instructions. The sample was analyzed double-blindly at Sun Biomedical Technology (Beijing) Co.Ltd. Plasma NT-proBNP concentrations were measured by electrochemical luminescence methods. Levels of creatinine, uric acid, white blood cell count and percentage of neutrophils were evaluated at the central chemistry laboratory of Peking University Third Hospital. Creatinine clearance was calculated according to the Cockcroft and Gaultequation (Matz2002).

### Echocardiography

Each patient underwent echocardiography lying in the left decubitus position at the time of study, entry using a GE-VingMedVechocardiographic machine (Vivid 7) with a 3.3-MHz multiphase array probe. The echocardiographic techniques and calculation of different cardiac dimensions and volumes were performed according to the guidelines of the American Society of Echocardiography (Schiller et al. 1989). Left ventricular ejection fraction (LVEF) was obtained using a modified biplane version of Simpson’s method with apical two- and four-chamber views. These studies were carried out by experienced cardiologists.

### Statistical Analysis

The Kolmogorov–Smirnov test was used to test for normal distribution of continuous variables. Variables with two groups were compared with the Student’s t-test. One-way ANOVA was used for comparing data for more than two groups; Student–Newman–Keuls method and the Tamhane method were used for homogeneous and non-homogeneous subsets, respectively. Proportions were compared using the χ2 test. Parametric correlation employed the Pearson correlation coefficient and the Spearman test was used for non-parametric correlation. Multiple linear regression analysis was used to identify factors that were independently associated with HSPs level. The cut-off concentration for HSPs was selected to yield the highest possible Youden Index score (sensitivity+specificity–1). Te area under the receiver-operating-characteristic (ROC) curve for HSPs was calculated. All analyses involved use SPSS 17.0 (SPSS Inc., Chicago, IL, USA). A p-value<0.05 (two-tailed) was considered statistically significant. Data were expressed numerically (as a percentage), as a median (interquartile range) or mean±standard deviation (SD) as appropriate.

## Results

### Isoproterenol Induced Cardiac Hypertrophy

**Figure 1:**
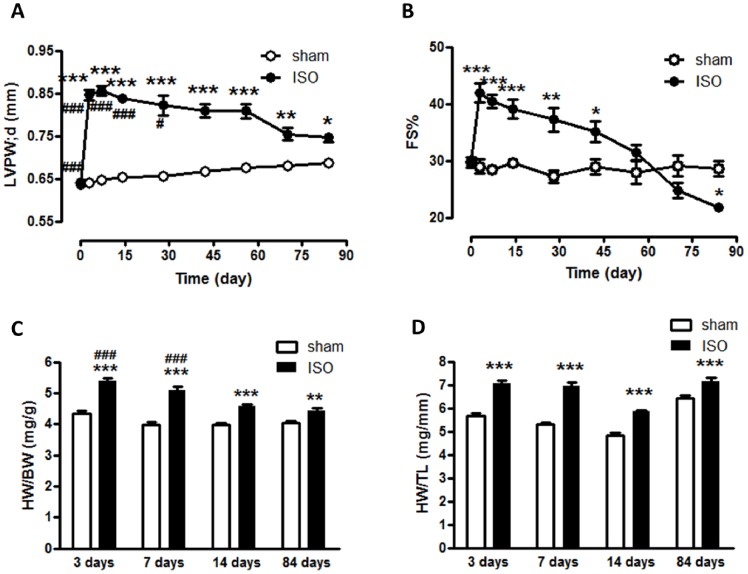
Isoproterenol induced cardiac hypertrophy and dysfunction in mice. (A). Left ventricular posterior wall thickness in end-diastolic (LVPW;d) significantly increased from 3 days to 84 days after ISO treatment; (B). Fractional shortening% (FS%) significantly increased during earlier stages upon ISO treatment, but reduced at 84 days, as compared with sham; (C) Ratio of heart weight to body weight (HW/BW) and (D) Ratio of heart weight to tibia length (HW/TL) of ISO groups was markedly higher than that of sham groups. *P<0.05, **P<0.01, ***P<0.001, compared with sham at the same time points. ^#^P<0.05, ^###^P<0.001, compared with ISO group at 84 days. ISO, isoproterenol.

**Figure 2:**
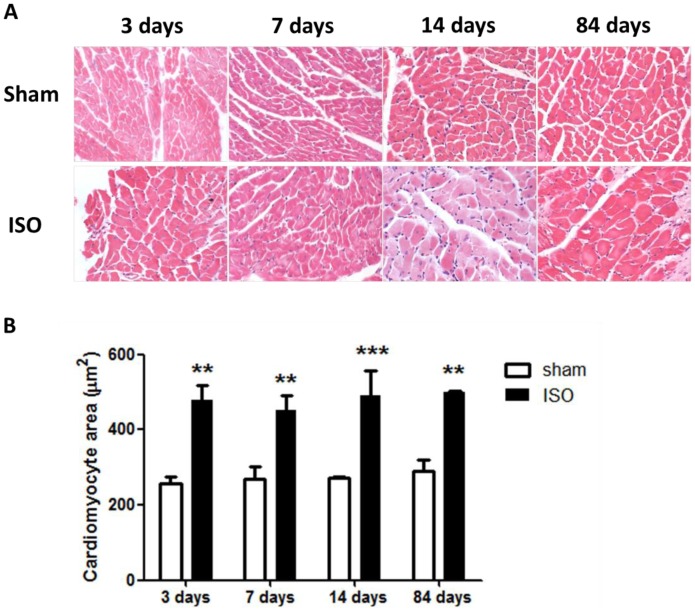
Isoproterenol induced cardiac hypertrophy as detected by HE staining. (A) HE staining of sections from the left ventricular myocardium of mice subjected to vehicle or isoproterenol. (B) Quantification of cross-sectional areas of the cardiomyocytes shown in (A). **P<0.01, ***P<0.001, compared with sham at the same time points. ISO, isoproterenol.

Mice receiving isoproterenol (ISO) infusion exhibited a significant increase in end-diastolic left ventricular posterior wall thickness (LVPW;d) compared with sham at 3 days (0.85±0.01 mm vs. 0.64±0.006 mm, P<0.001), 7 days (0.86±0.01 mm vs. 0.65±0.005 mm, P<0.001), 14 days (0.84±0.01 mm vs. 0.65±0.005 mm, P<0.001) and 84 days (0.75±0.01 mm vs. 0.68±0.006 mm, P<0.05, Figure 1A). In addition, the ratio of heart weight to body weight (HW/BW) and the ratio of heart weight to tibia length (HW/TL) in ISO groups were markedly higher than that in sham groups (Figure 1C and 1D), which also indicated that ISO induced substantial cardiac hypertrophy. Histological analysis showed that the size of ventricular myocytes increased significantly in mice receiving ISO (479.5±38.8 µm^2^ vs. 258.2±17.1 µm^2^ at 3 days; 452.5±38.8 µm^2^ vs. 268.7±32.0 µm^2^ at 7 days; 489.3±68.4 µm^2^ vs. 270.5±5.71 µm^2^ at 14 days; 498.4±5.48 µm^2^ vs. 289.2±30.1 µm^2^ at 84 days, Figure 2A and 2B), further demonstrating that cardiac hypertrophy occurred in response to ISO. As for the cardiac function, we observed that the mice subjected to ISO showed an increase in fractional shortening percentage (FS%) compared with sham group at 3 days (41.95±1.67% vs. 28.99±1.26%), 7 days (40.46±1.19% vs. 28.40±0.74%) and 14 days (39.04±1.67% vs. 29.55±0.75%), but a decline in FS at 84 days (21.83±0.66% vs. 28.59±1.33%) after ISO administration (Figure 1B), suggesting that heart failure occurred in this stage after infusion of ISO for 84 days.

### HSPs Selectively Increased during Isoproterenol-induced Cardiac Hypertrophy

**Figure 3:**
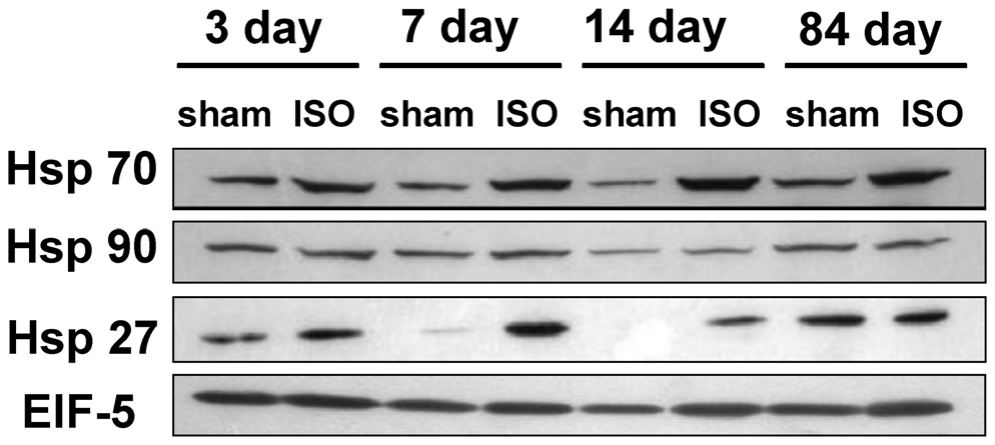
The expressions of HSP 70, HSP 90 and HSP 27 in the hearts from mice subjected to ISO or vehicle at 3days,7 days, 14days and 84 days. The levels of Hsp 70, Hsp 90 and Hsp 27 in hearts protein extracts were examined by Western blotting. A representative Western blot from three independent experiments is shown. EIF-5 was used as an internal control.

To investigate if the expression of HSPs changes during the periods of animal cardiac hypertrophy, we examined the expression level of HSPs with western blot analyses in mice cardiac hypertrophy model induced by ISO. The results showed that HSP27 and HSP70 time-dependently increased with cardiac hypertrophy but HSP90 did not changeat any stage (Figure 3).

### Only Plasma Concentration of HSP 70 Showed Positive Correlation to the Severity of Heart Failure in Patients

**Figure 4:**
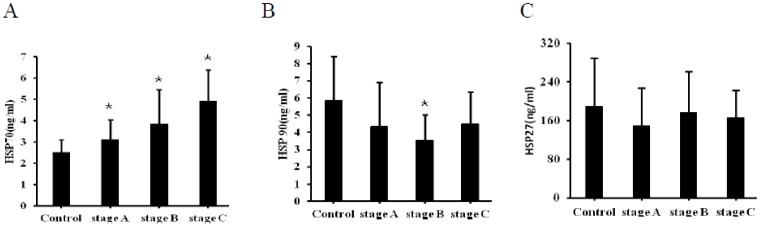
Plasma concentrations of HSP 70, HSP 90 and HSP 27 in different stages of heart failure compared with control subjects. (A), HSP 70 showed a stage-dependent increase in plasma concentration (*p<0.001). (B), Plasma concentrations of HSP90 decreased in stage B compared to the control group (*p<0.01), but didn’t change in stage A and C. (C), No statistically significant changes were found between each group as to plasma concentrations of HSP27.

Since HSP70 and HSP27 increased along with the progression of cardiac hypertrophy in mice, this gave rise to a question that if the level of HSPs showed a same profile during the process of heart failure in patients, so we collected blood samples from 38 control subjects and 184 patients with different stages of HF and detected the plasma concentrations of HSPs. Patients characteristics are summarized in Table 1. Stage C patients were more likely have a higher average heart rate, diuretic usage and uric acid level, and a lower creatinine clearance than the other three groups. Stage B patients had a history of diuretic usage than stage A and control groups. The total cholesterol, glycosylated haemoglobin and white blood cells were comparable in these groups. As shown in Figure 4, the plasma concentrations of HSP70 in patients with HF were not only much higher than the Cont group (2.52±0.59 ng/mL, P<0.01), but positively correlated to the severity of heart failure (stage A: 3.14±0.91 ng/mL, stage B: 3.86±1.61 ng/mL, stage C: 4.94±1.46 ng/mL, P<0.001). The correlation remained (r = 0.286,P<0.001) when the factors that could influence HSP70 concentrations were excluded, including age, diabetic mellitus status, uric acid level, creatine clearance and medication in multiple linear regression analysis. In contrast, no significant relationship was shown between HSP27, HSP90 and heart failure.

**Table 1:** Baseline clinical characteristics in different stages of heart failure.

Parameters	Control	Stage A	Stage B	Stage C	Total
	n = 38	n = 76	n = 68	n = 40	p-value
**Age(years)**	58.4±13.4#	66.1±11.2	67.2±10.3	70.4±12.4	0.001
**Sex(M/Total)**	52.63(20/38)	51.32(39/76)	55.88(38/68)	52.50(21/19)	0.320
**BMI(kg/m^2^)**	25.6±4.4	26.6±3.4	26.5±2.6	25.8±4.9	0.356
**CHD(Coronary heart disease)%**	0(0/38)#	55.3(42/76)	67.6(46/68)	45(18/40)	0.001
**Risk factor of coronary heart disease**					
**Smoking%**	31.6(12/38)	52.6(40/76)	51.4(35/68)	45(18/40)	0.743
**Hypertension%**	0(0/38)#	73.6(56/76)	80.9(55/68)	62.5(25/40)	<0.001
**Hyperlipidemia%**	0(0/38)#	43.4(33/76)	32.3(22/68)	22.5(9/40)	<0.001
**Diabetes mellitus %**	0(0/38)#	23.6(18/76)	23.5(16/68)	35(14/40)	0.001
**Medication**					
**Aspirin%**	5.2 (2/38)#	51.3(39/76)	54.4(37/68)	47.5(19/40)	<0.001
**ACEI/ARB%**	0(0/38)#	42.1(32/76)	50.0(34/68)	62.5(25/40)	<0.001
**β-Blockers%**	0 (0/38)#	38.1(29/76)	50.0(34/68)	52.5(21/40)	<0.001
**Statin%**	2.6(1/38)#	48.7(37/76)	48.5(33/68)	32.5(13/40)	<0.001
**Diuretics%**	0(0/38)	0(0/76)	11.7(8/68)*	95(38/40)*	<0.001
**Biochemical characteristics**					
**WBC(10^∧^9/L)**	5.81±1.84	6.17±1.47	6.56±1.72	6.43±1.71	0.589
**Neut(%)**	59.8±10.6	58.4±7.4	60.1±8.8	63.2±11.6	0.438
**CCr(ml/min)**	87.2±24.0	87.3±21.7	99.4±27.8	109.2±30.9*	0.004
**UA(umol/L)**	311.6±103.9	328.2±97.1	364.1±88.6	455.6±92.5*	0.001
**TC(mmol/L)**	4.16±0.81	4.57±1.01	4.46±1.12	3.98±1.26	0.156
**LDL(mmol/L)**	2.76±0.28	2.76±0.86	2.71±0.98	2.22±1.19	0.114
**HbA1c(%)**	5.95±0.21	6.34±1.09	6.75±1.33	6.47±1.56	0.874
**NT-proBNP(ng/L)**	44.6	92.3	288.9	3981.3*	0.010

Continuous variables expressed as mean±deviation (normal distribution) or medians with interquartile range (not normal distribution).

* Stage C patients had a higher average heart rate, diuretic usage, uric acid level and NT-proBNP than the other three groups. A lower creatinine clearance was also found in stage C.

* Stage B patients had a history of diuretic usage more than stage A and control groups.

# Control group population had a lower average age, and aspirin, ACEI/ARB, β-Blockers, statin usage than the other three groups and they had no history of hyperlipidaemia, hypertension and diabetes mellitus. CCr, creatinine clearance; UA, uric acid; TC, total cholesterol, HbAlC, glycosylated haemoglobin; NT-proBNP, amino-terminal pro-brain natriuretic peptide.

As to the echocardiographic parameters, plasma HSP70 was correlated with left atrial diameter (r = 0.202, p<0.05), left atrial pressure (r = 0.279, p<0.01). However, no relationship was found between HSP70 and left ventricular ejection fraction.

### HSP70 and NT-proBNP

**Figure 5:**
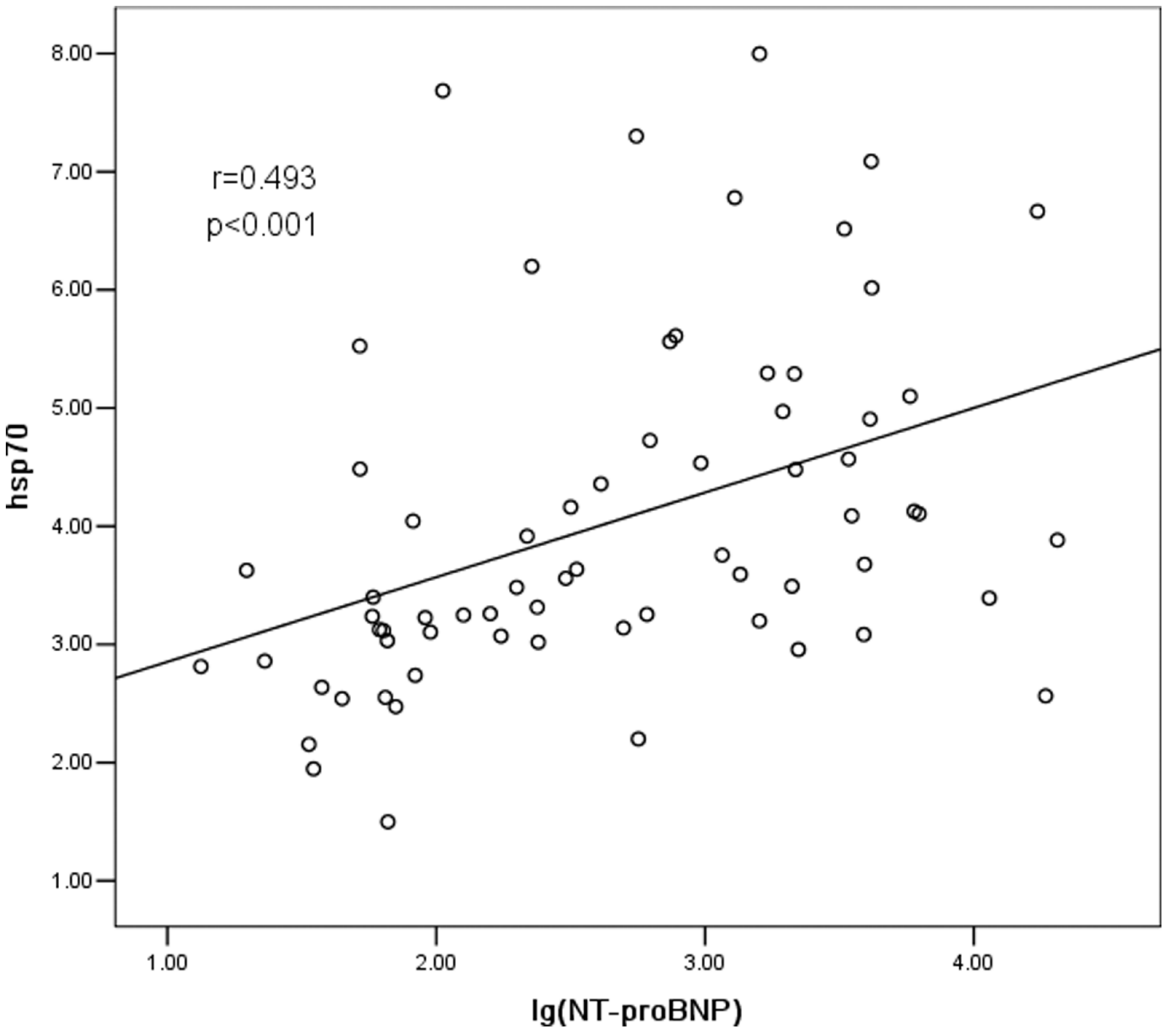
The correlation analysis between HSP70 and NT-proBNP. HSP70 was positively correlated to NT-proBNP (r = 0.493,P<0.001).

NT-proBNP is a well-accepted index that reflects left ventricular function. In the present study, the sensitivity and specificity of NT-proBNP and HSP70 were analyzed and compared. NT-proBNP showed positive correlation to the severity of heart failure (r = 0.794, P<0.001, data not shown). On another hand, the concentrations of HSP70 and NT-proBNP were also positively correlated (r = 0.493, P<0.001). The correlation remained (r = 0.252, P<0.05) still significant by excluding the confounding factors such as age and creatine when we excluded the factors such as age, creatine clearance. However, the correlation between them lost significance when the factor of heart failure stages was excluded (r = −0.112, P = 0.370), which implied that the relationship between NT-proBNP and HSP70 depended on the stage of heart failure (Figure 5). In other words, the identification of heart failure stage is very important to establish this relationship.

### The Receiver-operating Characteristic Curves

**Figure 6:**
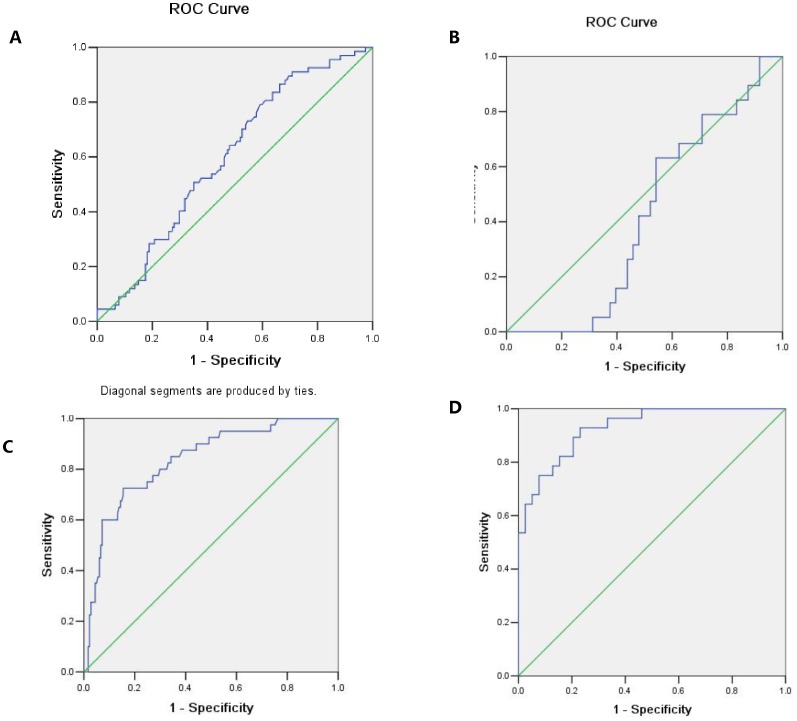
Receiver-operating characteristic curves to predict heart failure patients. (A), ROC curves of HSP70 for diagnosing stage B HF. (B), ROC curves of NT-proBNP for diagnosing stage B HF. HSP70 has a high sensitive cut-off value (2.72 ng/mL) in predicting stage B HF, while NT-proBNP hasn’t. (C), ROC curves of HSP70 for diagnosing stage C HF. (D), ROC curves of NT-proBNP for diagnosing stage C HF. NT-proBNP has a more sensitive cut-off value in diagnosing stage C HF (92.9%) while HSP70 has a more specific one (84.6%).

ROC curves of HSP70 or NT-proBNP diagnosing stage B or C of HF was investigated. ROC curves of HSP70 and NT-proBNP diagnosing stage B HF are shown in Figure 6A, B. The areas under curve (AUC) for HSP70 and NT-proBNP were 0.601 and 0.445, respectively. So as shown in Table 2, the odds ratio calculated for stage B HF in this study was 20.6 for HSP70, with the cut-off values at 2.72 ng/mL. Similarly, ROC curves of HSP70 and NT-proBNP diagnosing stage C HF are shown in Figure 6 C, D. The areas under curve (AUC) for HSP70 and NT-proBNP were 0.835 and 0.905. And as shown in Table 3, the cut-off value was 4.08 ng/mL for HSP70 and 324.5 pg/mL for NT-proBNP.

**Table 2:** Diagnostic value of heat shock protein 70 (HSP70) and amino-terminal pro-brain natriuretic peptide (NT-proBNP) in detecting stage B patients.

Factor	Cut-off value	AUC	SE	P	Youden	Sensitivity	Specificity
Hsp70	2.72 ng/mL	0.601	0.039	0.017	0.206	86.8%	33.8%
NT-proBNP	–	0.445	0.072	0.476	–	–	–

**Table 3:** Diagnostic value of heat shock protein 70 (HSP70) and amino-terminal pro-brain natriuretic peptide (NT-proBNP) in detecting stage C patients.

Factor	Cut-off value	AUC	SE	P	Youden	Sensitivity	Specificity
Hsp70	4.08 ng/mL	0.835	0.035	0.001	0.206	72.5%	84.6%
NT-proBNP	324.5 pg/mL	0.905	0.037	0.001	−0.275	92.9%	75%

## Discussion

It is well known that the level of HSPs is usually induced upon stresses. In the present study, we found that the expressions of HSP70 and HSP27 but not HSP90 increased during cardiac hypertrophy in mice, showing a selectively altering profile. In addition, the expressions of the two HSPs appeared to increase from the very early phase (at 3 days) in response to isoproterenol. The findings gave us a clue: the change in HSPs expressions may be developed as an effective index for early diagnosis of heart failure if it appears similar profile in patients. Thus, we then evaluated 222 patients with cardiovascular clinical or cardiographic findings. Except for control group, they were divided into three groups: (1) those at risk for development of clinical HF (such as coronary heart disease or high risk factor of coronary heart disease); (2) patients with cardiac structural abnormalities resulting from cardiac pathology (such as a previous myocardial infarction and left ventricular hypertrophy); and (3) patients with clinically identifiable HF. Patients were stratified according to ACC/AHA HF guidelines and the mean HSP level was then analyzed in this stratification. In our study, HSP70 level was positively correlated with the progression of HF. However, HSP27 and HSP90 were not observed related to HF.

HSPs, known as a family of intracellular proteins with cytoprotective function, were considered as molecular chaperones essential for cell survival both in physiological and stress conditions [14], [15]. Recently, HSP70 has got much attention as a clinical marker in heart failure when it was reported that increased circulating levels of HSP were associated with disease severity [16]. Stage B HF isn’t diagnosed as clinical HF because it does not show any specific symptoms that allow its identification during the early stages. Diagnosis is frequently made only after progression to later stages, at which point the deteriorating pathological prosess limits effective treatment. Therefore, the development of sensitive and specific methods for early detection has been a task of top priority for improving the treatment of this disease. It was observed that HSP70 progressively increased along with progression of the stages of HF (p<0.001), and the concentrations of HSP70 were already elevated at stage B HF patients compared with stage A (without echo-positive observation to find structural heart disease). This suggested that HSP70 concentrations may have potential clinical value in screening patients who do not have specific current clinical findings or history but may be progressing silently toward future HF.

HF is a progressive condition. In animal models overexpression of HSP70 protects the heart against damaging effects of ischemia [17]. Furthermore, it has been shown that stretch and decreased myocyte shortening results in an increased expression of HSP in the isolated perfused rabbit heart [15]. It is conceivable that HSP70 represents a protective mechanism not only in acute settings, e.g. associated with ischemia and reperfusion following coronary bypass grafting [18], but also in patients with chronic “stress”, as in CHF. It is reported that a reduction in the production of HSP70 may play a significant role in the decrease in contractile function during the development of heart failure in a rat model [19]. Increased expression of HSP70 could represent an innate protective mechanism which helped to restore physiological conditions [15]. It is known the activation of endogenous neurohormonal systems play an important role in cardiac remodeling and development into HF. It has been demonstrated in animal models that angiotensin II,isoporterenol and norepinephrine can induce production of HSP [20]–[22]. Plasma levels of epinephrine and norepinephrine are known to be significantly elevated in patients with CHF. Activation of the sympatho-adrenergic system in CHF could therefore explain the elevated levels of HSP70 seen in this condition. Despite its cytoprotective capacity, HSP70 activates the CD14/Toll-like receptor-4 receptor and induce the production of inflammatory cytokines, which can induce the synthesis of HSPs [23]. The relationship suggests that short-term cytokine activation is protective but during chronic activation, such as in CHF, it becomes detrimental even in patients with angiographically normal coronary arteries [24]. The source of circulating HSP in CHF is still unknown, but potentially it could be produced by white blood cells via activation of the CD14 receptor [18], the myocardium itself [25] or by the endothelium [26]. It was reported that intracellular and extracellular HSP70 have different roles in the regulation of cardiac remodeling and function in response to hypertension in pressure overload mice [27]. Thus, HSP70 participates in the whole pathophysiological progression of HF, and may acts as a clinical marker for early diagnosis. In contrast, although NT-proBNP has been used as a diagnostic and prognostic biomarker for a long time, when it was evaluated prospectively as a screening tool for stage B, the results were not promising [10], [11], and the guidelines did not define the use of NT-proBNP as a screening tool. In our study, the ROC curve analysis for diagnostic ability of stage B HF showed that HSP70 was more sensitive than NT-proBNP. Determining the concentrations of HSP70 and NT-proBNP may reflect the different pathophysiological aspects of HF and the combination might also facilitate the early detection of stage B to improve the management of HF.

In conclusion, based on our previous proteomic results, we have revealed that HSP27 and HSP70 were increased with the progression of cardiac hypertrophy and that HSP90 was not altered at the same time in animal cardiac hypertrophy model. In human the plasma concentration of HSP70 increased gradually with progression of ACC/AHA HF stages. However, HSP27 and HSP90 were not related to HF stages. HSP70 has potential implications for detecting stage B HF and monitoring high-risk HF patients.
